# High angular resolution diffusion imaging with stimulated echoes: compensation and correction in experiment design and analysis

**DOI:** 10.1002/nbm.3137

**Published:** 2014-06-03

**Authors:** Henrik Lundell, Daniel C Alexander, Tim B Dyrby

**Affiliations:** a Danish Research Centre for Magnetic Resonance, Centre for Functional and Diagnostic Imaging and Research, Copenhagen University Hospital HvidovreDenmark; b Centre for Medical Image Computing, Department of Computer Science, University College LondonGower Street, London, WC1E 6BT, UK

**Keywords:** diffusion MRI, diffusion tensor imaging, HARDI, STEAM, stimulated echo

## Abstract

Stimulated echo acquisition mode (STEAM) diffusion MRI can be advantageous over pulsed-gradient spin-echo (PGSE) for diffusion times that are long compared with *T*_2_. It therefore has potential for biomedical diffusion imaging applications at 7T and above where *T*_2_ is short. However, gradient pulses other than the diffusion gradients in the STEAM sequence contribute much greater diffusion weighting than in PGSE and lead to a disrupted experimental design. Here, we introduce a simple compensation to the STEAM acquisition that avoids the orientational bias and disrupted experiment design that these gradient pulses can otherwise produce. The compensation is simple to implement by adjusting the gradient vectors in the diffusion pulses of the STEAM sequence, so that the net effective gradient vector including contributions from diffusion and other gradient pulses is as the experiment intends. High angular resolution diffusion imaging (HARDI) data were acquired with and without the proposed compensation. The data were processed to derive standard diffusion tensor imaging (DTI) maps, which highlight the need for the compensation. Ignoring the other gradient pulses, a bias in DTI parameters from STEAM acquisition is found, due both to confounds in the analysis and the experiment design. Retrospectively correcting the analysis with a calculation of the full *B* matrix can partly correct for these confounds, but an acquisition that is compensated as proposed is needed to remove the effect entirely.

## INTRODUCTION

Stimulated echo acquisition mode (STEAM) diffusion MRI 1,2 offers advantages over the more common pulsed-gradient spin-echo (PGSE) diffusion MRI when *T*_2_ is short compared with the diffusion time and *T*_1_ ≫ *T*_2_. The STEAM signal decays with rate *T*_1_ during the mixing time *τ*_m_, which determines the diffusion time, whereas *T*_2_ decay occurs throughout the whole PGSE sequence. PGSE is preferred for many standard diffusion-weighted imaging (DWI) applications and avoids the initial 50% signal loss inherent in STEAM, where half of the echo is lost. However, STEAM diffusion MRI is common in tissue with short *T*_2_, such as muscle or cartilage 3. STEAM is also used in diffusion-weighted spectroscopy (DWS) of metabolites with short *T*_2_ 4. STEAM is also useful for measuring diffusivity in water compartments with significantly lower *T*_2_, like myelin water, as previously shown in preclinical settings with PGSE 5. In general, *T*_2_ decreases and *T*_1_ increases as field strength increases and early evidence 6 suggests benefits of STEAM for *in vivo* human-brain diffusion tensor imaging (DTI) at 7T. *Ex vivo q*-space studies of brain tissue 7–9 usually prefer STEAM over PGSE, as fixation and lower temperature reduce diffusivity compared with *in vivo* studies, which increases the necessary diffusion times 10,11. Similarly, Dyrby *et al.* 12 demonstrated the need for long diffusion times to ensure sensitivity to large axon diameter, which is important for microstructure imaging techniques such as ActiveAx 13 and AxCaliber 8.

Although STEAM offers benefits over PGSE in obtaining long diffusion times when *T*_2_ relaxation is short, gradients other than the diffusion gradients can significantly degrade its potential by introducing a directional diffusion-weighting bias, if not taken into account. The most significant contributions are usually from the crusher and slice-select gradients, here referred to as butterfly gradients, which add unwanted diffusion-weighting ‘cross terms’ in the *B* matrix 14. In PGSE, their contribution to diffusion weighting is usually negligible in practice, because the diffusion time for the butterfly gradients is only a few milliseconds (the length of the refocusing pulse). In STEAM, that contribution is typically much more significant, because the butterfly gradients are separated by the diffusion time, approximately *τ*_m_, and that is often much longer than for PGSE. Nevertheless, previous work with STEAM diffusion MRI, such as 8,9, follows standard practice for PGSE and neglects this effect. Mattiello *et al*. 15 derived the full *B* matrix accounting for all gradient pulses, which predicts the signal exactly on the assumption of Gaussian dispersion, i.e. the diffusion tensor model. However, additional diffusion weighting from other pulses skews the effective diffusion direction, disrupting the experimental design, i.e. the even distribution and strength of the sampled gradient directions usually acquired in DTI and high angular resolution diffusion imaging (HARDI) techniques. This is a critical issue in brain imaging, where diffusion is often strongly anisotropic and non-uniform gradient orientations can lead to significant orientational bias in the precision of derived diffusion metrics 16.

In this article, we propose a simple compensation for the STEAM sequence, referred to as *compensated* acquisition, that accounts for the unwanted directional bias caused by the butterfly gradients. The implementation of the compensated acquisitionis simple and only requires a correction of the gradient vectors loaded on the scanner. We show how this compensation effectively cancels the effects of the butterfly gradients, so that the resulting data sets can be treated as if they came from an idealized HARDI protocol, i.e. ignoring the butterfly gradients. We demonstrate the need and effectiveness of the compensation for STEAM through HARDI–DTI experiments in simulation and on data acquired from a fixed monkey brain. Numerical experiments show that ignoring the butterfly gradients in STEAM leads to severe bias in the fitted diffusion tensor and derived quantities. The full *B*-matrix formulation from 15 reduces the bias, but some still remains because of the disruption to the intended even distribution of gradient directions. However, the compensated acquisition reduces bias even further and simplifies the calculation by allowing us to ignore the cross terms in the *B* matrix.

## METHODS

### STEAM pulse sequence

**Figure 1:**
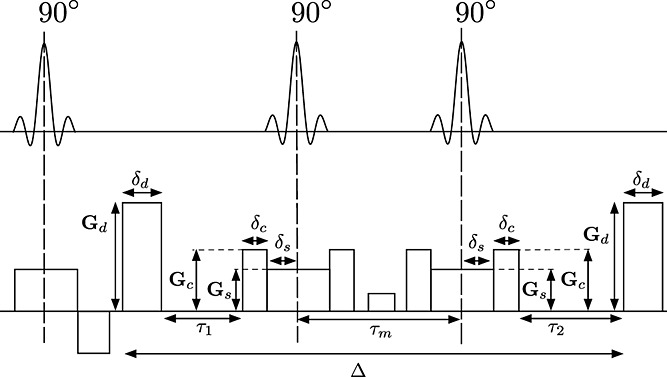
Diagram of the Stimulated Echo Aqcuisition Mode (STEAM) pulse sequence. STEAM resembles conventional Pulsed Field Gradient Spin Echo (PGSE) sequences, but the refocusing pulse is devided into two 90° pulses, which store the magnetisation along the longitudinal axis during the time *τ*_m_. During this time, the signal is subjected to *T*_1_ relaxation, which is normally much slower than the *T*_2_ relaxation in the transversal plane. This allows longer gradient separation, Δ, and thus longer effective diffusion times. In this study we consider the diffusion weighting from the diffusion encoding gradient G_d_, the crusher gradient G_c_ and the slice gradient G_s_ (the latter two referred to as the butterfly gradients), with respective lengths δ_d_, δ_c_ and δ_s_. With long *τ*_m_, the diffusion weighting from the butterfly gradients can be significant. This weighting causes biases, but its effect can be compensated for by adjusting G_d_.

The compensation in subsequent sections assumes the idealized STEAM pulse sequence in Figure 1. We refer to this figure for nomenclature. The layout is very similar to the conventional PGSE sequence, with diffusion-encoding gradients **G**_d_ on each side of the refocusing pulse. All gradients working on the signal pathway in the transversal plane introduce a diffusion weighting. In our case, the major sources, in addition to **G**_d_, are a crusher pulse **G**_c_ and a slice selection pulse **G**_s_. For a conventional sinc RF pulse, the phase modulation corresponds to half the area of the slice selective gradient. In a practical imaging set-up, the crusher, or the same effect of the diffusion encoding gradient, is also needed to isolate the original coherence pathway 17. We will refer to data with |**G**_d_| = 0 as nominal *b* = 0 measurements.

### Diffusion tensor imaging and STEAM

On the assumption of zero-mean Gaussian particle dispersion, i.e. the diffusion tensor (DT) model, the general formula 18:

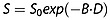
1predicts the signal, where *B* = *γ*^2^ ∫ **F**(*t*)**F**^T^(*t*)d*t* is the *B* matrix 14,

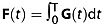
2

**G**(*t*) is the effective gradient vector at time *t*, · is the matrix scalar product, *D* is the DT, *γ* is the gyromagnetic ratio, 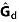 is a unit vector in the direction of **G**_d_ and *S*_0_ is the signal with *b* = 0. A common approach is to assume that **G**_c_ and **G**_s_ are negligible, so that we need only consider **G**_d_, which reduces Equation [1] to

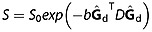
3where

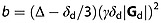
4and

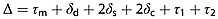
5is the separation between the diffusion gradients in the STEAM sequence. However, including the contribution from all relevant gradients in Figure 1 gives

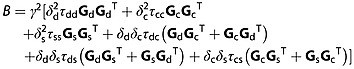
6where

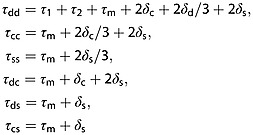
7

The expression in Equation [6] is the sum of pairwise interactions between the diffusion, crusher and slice-select pulses, similar to the *B* matrix for PGSE in 15.

### Compensated acquisition correction

The goal of the compensation is to cancel out the effect of the butterfly gradients by modifying the diffusion gradient directions. A simple correction finds the adjustment of **G**_d_ that minimizes the diffusion weighting of the nominal *b* = 0. For intended gradient vector **G**_i_, we instead acquire **G**_d_, which with the contributions from **G**_s_ and **G**_c_ produces the approximated *effective gradient vector* **G**_d_′ close to **G**_i_. The simple compensation we propose replaces each **G**_d_ in the list of gradient vectors we intend to acquire with

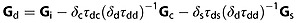
8

Equation [8] comes from setting **G**_d_ = *g***G**_c_ + *h***G**_s_ and minimising the trace of the *B* matrix in Equation [6] with respect to *g* and *h* to obtain *g* = − *δ*_c_*τ*_dc_(*δ*_d_*τ*_dd_)^− 1^ and *h* = − *δ*_s_*τ*_ds_(*δ*_d_*τ*_dd_)^− 1^. The weightings *h* and *g* depend only on the timings of the pulses so are constant within one HARDI shell, but may vary between shells or measurements with different *b* value or diffusion time. Another choice of **G**_d_ is 
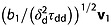
, where **v**_1_ is the primary eigenvector of the *B* matrix and *b*_1_ is the corresponding eigenvalue. However, the two choices for **G**_d_ are very similar in practice. The former is simpler to compute, while the latter applies to more general gradient configurations where the *B* matrix is more easily assessed by numerical integration.

In practice, implementation of the compensated acquisition simply requires an adjustment to the gradient direction scheme and corresponding *b* values or gradient strengths uploaded to the scanner. Precise implementations may differ among vendors. Also note that the gradient strength after compensation may exceed the maximum available gradient strength. A solution to this is simply to negate the direction of the intended direction, as the compensation is an additive vector.

### Post-processing correction

We consider three approximations to the signal that account for the butterfly gradients in different ways.

- Approximation 1 (A1) is the approach generally used in DTI analysis of PGSE. It ignores butterfly gradients and considers only diffusion gradients.
- Approximation 2 (A2) accounts for the butterfly gradients in a simple but non-trivial way by identifying an effective diffusion gradient **G**_d_′ that incorporates the diffusion weighting of the diffusion and butterfly gradients. Our choice comes from inverting Equation [8]:

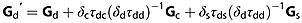
9

•Approximation 3 (A3) calculates the full *B* matrix as in Equation [6] and accounts for cross terms that A2 does not.

A1 also uses Equation [3] directly to fit the diffusion tensor. A2 also uses Equation [3], but with **G**_d_′ from Equation [9] replacing **G**_d_.

A3 uses the full *B* matrix, analogous to 15 for PGSE, rather than the single *b* value and gradient direction in A1 and A2.

By assuming a single *b* value, A1 and A2 ignore the cross terms in the *B* matrix, which express the interaction between temporally separated gradient components with different orientation 15. A1 is exact only when **G**_c_ = **G**_s_ = 0. A2 is exact only when **G**_d_, **G**_c_ and **G**_s_ all have the same orientation. A3 accounts for all cross terms, so is always exact for the Gaussian dispersion assumed in DTI. In the absence of butterfly gradients, A3 and A2 are equivalent to A1. An overview of the parameter definitions and signal models is given in Table1.

**Table 1:** Short descriptions of (a) gradient definitions used in Equations [2]–[9] and (b) signal models used in the analysis

Name	Description
(a)	
**G**(*t*)	The effective gradient time vector including all gradients.
**G**_d_	The applied diffusion encoding gradient vector.
**G**_c_	The crusher gradient vector.
**G**_s_	The slice gradient vector.
**G**_i_	The intended diffusion encoding gradient vector.
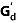	The effective gradient vector that with the contributions from **G**_c_ and **G**_s_ approaches **G**_i_ after compensation.
(b)	
A1	Assuming diffusion weighting from **G**_d_.
A2	Assuming diffusion weighting from 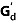 .
A3	Full *B*-matrix calculated from **G**_d_, **G**_s_ and **G**_c_.

## EXPERIMENTS

### DWI protocols

The experiments compare three kinds of imaging protocol: (i) STEAM protocol, which does not make the adjustment in Equation [8] but uploads the intended list of directions and *b* values to the scanner; (ii) compensated STEAM acquisition (STEAMCOMP) protocol, which does include the adjustment in Equation [8]; and (iii) PGSE protocol. The PGSE was included to provide principal direction estimates minimally affected by the butterfly gradients for comparison with those from STEAM. All protocols are representative of real-world applications and were in fact optimized for axon diameter mapping of post-mortem brain tissue using ActiveAx; see 19 for details. For the PGSE, two protocols were chosen with high and low gradient strength. Table 2 shows the settings for the preclinical protocols used.

**Table 2:** (a) PGSE and (b) STEAM protocols. Both come from the experiment design optimisation in 12,25 with G_max_ = 300 mTm^− 1^. *N* is the number of diffusion-weighted images in each shell. *K* is the number of nominal *b* = 0 images associated with each shell. The nominal *b* = 0 images in STEAM have the same τ_m_ as the diffusion-weighted images. The compensated STEAM protocol STEAMCOMP follows (b), but replaces each G_d_ according to Equation [8]. Please refer to Figure 1 for notation

*N*	*K*	|G_d_|/mTm^− 1^						
(a)			Δ/ms	*δ*_d_/ms		*b*/smm^− 2^	*τ*_e_/ms	*τ*_r_/ms
103	25	300.0	12.9	5.6		2243	36.8	2600
106	25	219.2	20.4	7.0		3084	36.8	2600
(b)			*τ*_m_/ms	*δ*_d_/ms	*τ*_1_/ms	*b*/smm^− 2^	*τ*_e_/ms	*τ*_r_/ms
108	25	113.5	137.0	5.0	3.4	3425	26.0	2600

Every image in STEAM and STEAMCOMP has *δ*_c_ = 1.5ms, **G**_c_ = (0, 0, 0.15)Tm^− 1^, *δ*_s_ = 1.0ms, **G**_s_ = (0, 0, 0.15)Tm^− 1^ and *τ*_2_ = 0.

Since **G**_c_ and **G**_s_ are both along the slice direction (0, 0, 1), the compensation **G**_i_ − **G**_d_ from Equation [8] is along the negative slice direction; |**G** − **G**_d_| = 68.5mTm^− 1^.

### Imaging experiments

We acquire data from a fixed monkey brain, prepared as in 10. The live monkey was handled and cared for on the Island of St Kitts according to a protocol approved by the local ethics committee (The Caribbean Primate Center of St Kitts). Data were acquired on a Varian 4.7T preclinical scanner using a quadrature volume coil. All datasets were acquired at 0.5^3^mm^3^ isotropic resolution with a 256 × 128 matrix with 15 contiguous sagittal slices including the mid-sagittal plane.

### Simulation experiments

The simulation experiments use two DTs, one with eigenvalues {0.6, 0.2, 0.2} × 10^− 9^m^2^s^− 1^, which are typical of coherent white matter in fixed brain tissue at this *b* value, and the other {0.4, 0.4, 0.4} × 10^− 9^m^2^s^− 1^, which is isotropic with the same trace. The anisotropic DT has two variations: the first has principal eigenvector **e**_1_ = {0, 0, 1}, so that **G**_c_ and **G**_s_ are parallel to the fibre direction, and the second has **e**_1_ = {1, 0, 0}, so they are perpendicular.

With sufficient diffusion weighting, **G**_d_ can perform the function of the crusher gradients, so that **G**_c_ can be set to zero for all but the nominal *b* = 0 images. This may reduce the need for the proposed commensuration, although **G**_s_ necessarily remains non-zero. To test the need for compensation in such experiments, additional simulations were thus performed with **G**_c_ = {0, 0, 0}.

Each experiment adds Rician noise, so that the signal-to-noise ratio of the unweighted signal is 20. Weighted linear least-squares fitting 20 estimates the DT using each approximation from which we compute the eigenvalues, fractional anisotropy (FA) and **e**_1_. We repeat the procedure over 10 000 independent noise trials and compute the mean and standard deviation of the largest eigenvalue λ_1_ and the FA. We also compute the mean angle *α* between the estimated and true **e**_1_ for the anisotropic DTs. For all DTs, we compute the direction concentration *γ* = − *log*(1 − *E*), where *E* is the largest eigenvalue of the mean dyadic tensor 21. The direction concentration is zero for an isotropic set of directions and increases as the variance of the distribution decreases, reaching infinity when all align perfectly. Typical values of γ for similar noise trials with anisotropic tensors in 21 are 6–8. Unbiased noise trials with the isotropic tensor should produce γ close to zero.

To give some idea of the significance of the effects in a human imaging protocol, we repeat the experiment using *in vivo* settings for a clinical scanner. The protocol has seven nominal *b* = 0 images and 60 gradient directions with *b* = 1085smm^− 2^, |**G**_d_| = 40mTm^− 1^, *τ*_m_ = 300ms, *δ*_d_ = 5.5ms, *τ*_1_ = *τ*_2_ = 0, *δ*_c_ = 3ms, *δ*_s_ = 1.5ms, **G**_c_ = {10, 0, 0}mTm^− 1^ and **G**_s_ = {10, 0, 0}mTm^− 1^. The butterfly gradients are weaker than for the *ex vivo* protocol, because the voxel size is larger (2 mm isotropic). The test DTs have eigenvalues {1.7, 0.2, 0.2} × 10^− 9^m^2^s^− 1^ and {0.7, 0.7, 0.7} × 10^− 9^m^2^s^− 1^.

### Data analysis

We fit the DT to the STEAM, STEAMCOMP and PGSE data using weighted linear least-squares and construct colour-coded FA maps 22. We quantify the orientational similarity between pairs of DT volumes by computing the mean over the brainof the absolute dot product of principal directions weighted by DT linearity 23.

## RESULTS

### HARDI gradient direction file

**Figure 2:**
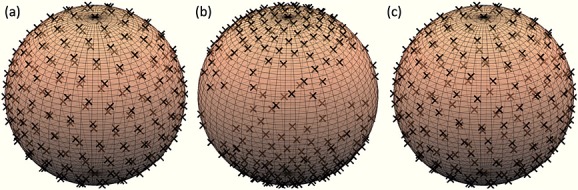
Illustration of the target and effective gradient directions in the STEAM protocols using the 108 directions in the STEAM protocol. A black cross marks each direction; shaded crosses are on the far side of the sphere. Panel (a) shows the target set with a cross in both positive and negative gradient directions. Panel (b) shows the set of effective gradient directions, i.e. the direction of G_d_′ in Equation [9], without compensation (STEAM); they skew strongly towards the slice direction. Panel (c) shows the effective gradient directions after compensation (STEAMCOMP); these are close to the target set.

Figure 2 shows the distribution of effective gradient directions, i.e. the orientation of **G**_d_′ from Equation [9], for STEAM and STEAMCOMP to illustrate the disruption to the HARDI design. Without compensation, the butterfly gradients skew the effective gradient directions strongly towards the slice direction. The true *b* values of the uncompensated gradient scheme ranged from 660–8830 s mm^-2^ with a maximum deflection from the intended direction of 36° and the mean deflection over all directions was 22°. With the crusher gradients turned off, the *b* values still ranged from 2075–5160s mm^−2^ with a maximum deflection of 13° and the mean deflection over all directions was 9°. The compensated protocol has evenly distributed effective gradient directions and a constant *b* value.

The nominal *b* = 0 were not compensated. As an indication of the impact of butterfly gradients, the *b* value from them alone, as in the nominal *b* = 0, is 1316s mm^− 2^ for the preclinical STEAM protocol. The same figure for the PGSE protocol is 17 smm^− 2^.

### Simulation experiments

Tables 3 and 4 list statistics for the fixed-tissue simulations with anisotropic and isotropic DTs, respectively. We show the two extreme cases, when the principal eigenvector of the DT is perpendicular and parallel to the slice direction. Note that perfect compensation makes A1 and A2 equivalent. Without compensation, A1 shows significant bias in FA, λ_1_ and **e**_1_ with both orientations of the anisotropic DT. Bias is most severe for **e**_1_ parallel to the butterfly gradients. In this case, the nominal *b* = 0 acquisition has higher diffusion weighting and larger signal attenuation than the intended diffusion-weighted signal. Here, DT estimation with A1 completely fails, producing negative eigenvalues and artificially high FA.

**Table 3:** Statistics from simulations with anisotropic DTs for each approximation using the preclinical STEAM protocol and a SNR of 20. Two simulated datasets were created, one with the DT perpendicular (⊥) and one with the DT parallel (∥) to the butterfly gradient direction. The units of λ_1_ are 10^− 10^m^2^s^− 1^; the units of α are degrees. The true FA is 0.603 and the true λ_1_ is 6 × 10^− 10^m^2^s^− 1^. Higher γ is better in this experiment

Gradients	Uncompensated						Compensated			
Analysis	A1		A2		A3		A1/A2		A3	
DT orientation	⊥	∥	⊥	∥	⊥	∥	⊥	∥	⊥	∥
FA	0.514	0.884	0.573	0.495	0.573	0.495	0.575	0.574	0.575	0.574
std	0.035	0.166	0.025	0.042	0.025	0.042	0.020	0.021	0.020	0.021
λ_1_	5.518	1.522	5.278	3.377	5.278	3.377	5.566	5.544	5.566	5.544
Std	0.262	0.270	0.212	0.185	0.212	0.185	0.161	0.165	0.161	0.165
*α*	4.670	63.714	2.537	5.058	2.537	5.058	1.946	1.992	1.946	1.992
γ	5.309	1.909	6.238	5.362	6.238	5.362	6.766	6.719	6.766	6.719

**Table 4:** Statistics, as in Table 3, from simulations with isotropic DTs. The true FA is 0; the true λ_1_ is 4 × 10^− 10^m^2^s^− 1^. Here γ should be zero

Gradients	Uncompensated			Compensated	
Analysis	A1	A2	A3	A1/A2	A3
FA	0.283	0.175	0.175	0.058	0.058
std	0.074	0.033	0.033	0.019	0.019
*λ*_1_	3.806	3.658	3.658	4.023	4.023
std	0.305	0.171	0.171	0.103	0.103
*γ*	1.504	0.846	0.846	0.414	0.414

Estimates of the isotropic DT show artefactual non-zero FA and significant direction concentration: *γ* = 1.5 means 95% of directions are within 6° of the mean. Compensation dramatically improves A1. Some downward bias remains in both FA and λ_1_ of the anisotropic DTs, but the bias is similar for both orientations. Compensation largely removes artefactual non-zero FA and orientational bias in the isotropic DT estimates: *γ* = 0.4 is typical for a uniformly distributed random sample of 10 000 directions and the 95% angle is over 25°.

Without compensation, A2 and A3 produce very similar results. Both significantly reduce bias compared with A1, although bias remains orientationally dependent and is strongest with parallel **G**_c_ and **G**_s_. Compensation reduces the bias and variance of parameter estimates from A3, especially for parallel **e**_1_, and removes orientational dependence. With compensation, A3 shows no benefit over A1 or A2. Tables 5(a) and (b) are equivalent to Tables 3 and 4, but with the crusher gradients turned off. The biases before compensation are smaller but still significant and compensation improves consistency and reduces orientational dependence.

**Table 5:** Statistics from simulations for each approximation using the preclinical STEAM protocol for (a) anisotopic DT and (b) isotropic DT. The experiments are similar to the imaging and simulation experiments presented in Tables 3 and 4, but with the crusher gradients turned off. The biases before compensation are only caused by the slice gradient and thus smaller, but consistency is still improved after compensation

Gradients	Uncompensated						Compensated			
Analysis	A1		A2		A3		A1/A2		A3	
(a)										
DT orientation	⊥	∥	⊥	∥	⊥	∥	⊥	∥	⊥	∥
FA	0.498	0.671	0.575	0.534	0.575	0.534	0.577	0.576	0.577	0.576
std	0.024	0.025	0.021	0.024	0.021	0.024	0.020	0.020	0.020	0.020
*λ*_1_	5.576	6.426	5.512	5.071	5.512	5.071	5.579	5.574	5.579	5.574
std	0.181	0.333	0.173	0.175	0.173	0.175	0.160	0.162	0.160	0.162
*α*	2.602	5.874	1.982	2.451	1.982	2.451	1.889	1.922	1.889	1.922
*γ*	6.212	6.260	6.730	6.351	6.730	6.351	6.825	6.790	6.825	6.790
(b)										
FA	0.210		0.074		0.074		0.057		0.057	
std	0.032		0.023		0.023		0.018		0.018	
*λ*_1_	5.168		3.981		3.981		4.025		4.025	
std	0.212		0.119		0.119		0.099		0.099	
*γ*	3.843		0.612		0.612		0.415		0.415	

Tables 6 and 7 show the corresponding results from the *in vivo* human protocol. Without compensation, A1 still produces considerable bias, which A2 or A3 reduces. The compensation provides only minor further improvements with A3 compared with A2.

**Table 6:** Simulation statistics for anisotropic diffusion with the human protocol. Two simulated datasets were created, one with the DT perpendicular (⊥) and one with the DT parallel (∥) to the butterfly gradient direction. The true FA is 0.87 and the true λ_1_ is 17 × 10^−10^ m^2^ s^−1^

Gradients	Uncompensated						Compensated			
Analysis	A1		A2		A3		A1/A2		A3	
DT orientation	⊥	∥	⊥	∥	⊥	∥	⊥	∥	⊥	∥
FA	0.899	0.859	0.861	0.864	0.861	0.864	0.863	0.863	0.863	0.863
std	0.019	0.017	0.018	0.015	0.018	0.015	0.017	0.017	0.017	0.017
*λ*_1_	14.807	16.203	15.928	16.260	15.928	16.260	16.263	16.269	16.263	16.269
std	0.581	0.533	0.610	0.548	0.610	0.548	0.532	0.521	0.532	0.521
*α*	19:628	3:862	1.502	1.493	1.502	1.493	1.391	1.390	1.391	1.390
*γ*	7.132	7.380	7.288	7.300	7.288	7.300	7.437	7.437	7.437	7.437

**Table 7:** Simulation statistics for isotropic diffusion with the human protocol. The true FA is 0 and the true λ_1_ is 7 × 10^− 10^m^2^s^− 1^

Gradients	Uncompensated			Compensated	
Analysis	A1	A2	A3	A1/A2	A3
FA	0.356	0.098	0.098	0:095	0.095
std	0.042	0.033	0.033	0.031	0.031
*λ*_1_	5.433	4.313	4.313	4.311	4.311
std	0.305	0.254	0.254	0.238	0.238
*γ*	3.852	0.462	0.462	0.414	0.414

### Imaging experiments

**Figure 3:**
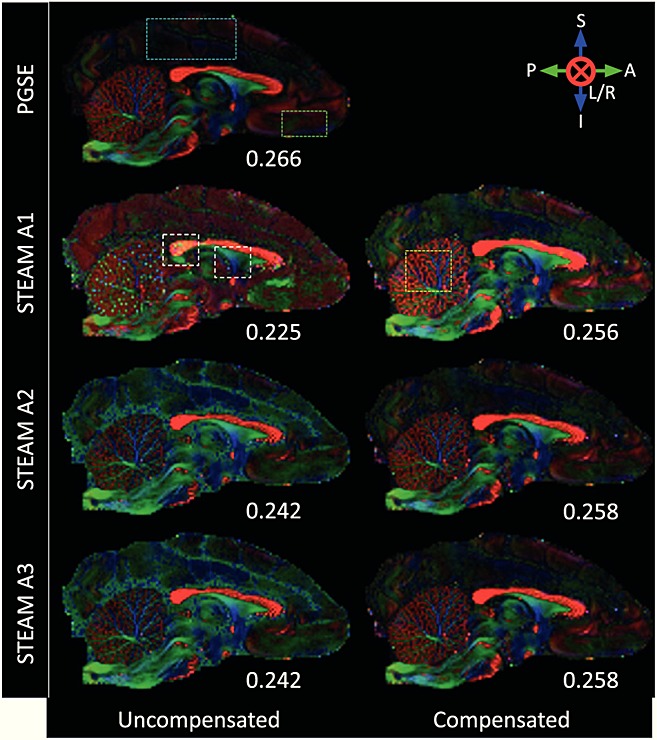
Direction-encoded colour maps 22 for the mid-sagittal slice of the monkey brain from the b = 2243 s mm^− 2^ shell of PGSE (top left), STEAM (left) and STEAMCOMP (right). Rows 2–4 show the maps reconstructed with A1, A2 and A3, respectively. The numbers quantify the orientational similarity (definition in the text) between each map and the b = 3084 s mm^− 2^ shells of PGSE.

Figure 3 compares maps from PGSE with STEAM for each approximation qualitatively. The number next to each STEAM map is the orientational similarity with the *b* = 3084 s mm^− 2^ shell PGSE; higher numbers show greater agreement. The number next to the PGSE map is the orientational similarity of the *b* = 2243 s mm^− 2^ and *b* = 3084 s mm^− 2^ shells of PGSE.

For STEAM, A1 introduces upward bias in FA in the superior half of the brain, where diffusion should be close to isotropic, such as the area in the cyan box on the PGSE map. The maps also show orientation bias towards the left–right slice direction (the map appears red) due to the directional bias towards the direction of the butterfly gradients shown in Figure 2. The white boxes show bias in anisotropic regions: the left box shows severely biased orientation estimates (some voxels appear green rather than red) in the corpus callosum, where the fibres are parallel to the butterfly gradients; the right box shows less biased orientation estimates in the fornix, which has perpendicular fibres. A2 and A3 are qualitatively indistinguishable from one another and are more consistent with the PGSE map than A1, e.g. in the white boxes. However, they still show upward bias in FA together with consistent artefactual orientation in isotropic regions in the cortex(blue/green colour in cyan box region). Two directions exceeded *G*_max_ after compensation and were truncated to *G*_max_ by the scanner. This imperfection in compensation leads to the difference between the compensated results in A1 versus A2 and A3.

## DISCUSSION

For some DWI applications with long diffusion times compared with the *T*_2_ of the tissue, STEAM provides advantages over the more commonly used PGSE. This article highlights the need to account for the undesired diffusion weighting of slice and crusher gradients, i.e. butterfly gradients, in STEAM diffusion MRI. This additional diffusion weighting is not problematic in PGSE, but in STEAM it adds a directional bias that disrupts the assumed uniform gradient directions and strengths. If ignored, the additional diffusion weighting in STEAM leads to bias in the parameter estimates in two ways: model inaccuracy and disrupted experiment design. Model inaccuracy can be ameliorated by using the full *B* matrix (model A3 here) or approximately using the effective gradient (model A2). However, bias from the disrupted experiment design, i.e. non-uniform gradient directions and strengths, remains. The compensation we propose here, which occurs during the acquisition stage by modifying the diffusion gradient vectors in the scheme file used by the scanner, ameliorates the disrupted experimental design avoiding the additional bias. The size of the improvement depends largely on the experimental settings, as seen in the realistic examples in our real and simulated experiments, but the compensation itself works for any choice of parameters. Thus, we recommend that the design of STEAM experiments is always compensated in this way at the acquisition stage. The compensation can be computed both analytically for a standard STEAM set-up, as in our case, or by optimizing the desired *B* matrix by numerical integration of any arbitrary gradient configuration.

Simulation and fixed-brain data experiments demonstrated that, without the compensation and using the standard DTI model (A1 uncompensated), severe bias can arise in estimated DTI parameters. Accounting for cross terms in the *B* matrix either exactly (A3 uncompensated) or approximately (A2 uncompensated) reduces that bias considerably; the lack of performance difference between A2 and A3 shows that the minor eigenvalues of the *B* matrix are negligible. However, including the compensation at acquisition time results in further reductions in bias (e.g. A3 compensated versus A3 uncompensated), highlighting the importance of the compensation. Differences in results from A3 with and without compensation at acquisition show the effect of the experiment design disruption, which is seen in both simulation and real data. The disruption to the experiment design affects parameter estimates most strongly with parallel diffusion and butterfly gradients, because the additional diffusion weighting in the fibre direction pushes parallel signals further into the noise floor. The effect for an arbitrary tensor orientation is in between the two extreme cases explored by the simulation with the tensor aligned parallel or perpendicular to the slice direction. Compensation removes the experiment design disruption and thereby improves the precision in parameter estimates and removes the orientational dependence. Moreover, A1, A2 and A3 after compensation provide very similar results, showing that the compensated acquisition largely eliminates the cross terms in the *B* matrix, thus enabling the use of A1. This is a significant advantage, as A1 is simple to implement and compute and is used in the majority of DTI and HARDI reconstruction software tools. A2 and A3 are useful in their own right for analysis of existing data without the compensation. In addition to HARDI techniques, single-direction model-based STEAM diffusion MRI applications, such as 8,9, are also likely to benefit significantly from the proposed compensation.

In simulations we observe a bias in FA and λ_1_ from A3 with compensation, which is unavoidable, since the model is exact and the experiment design is not disrupted. It comes from Jones' ‘squashed-peanut’ effect 20: a Rician noise effect as measurements with gradient parallel to **e**_1_ approach the noise floor.

The performance differences are less marked in the human protocol, because the butterfly gradients are smaller due to the lower resolution. However, the values of *α* between 3.8° and 19.6° that we observe for A1 without compensation are at least as large as the orientational bias incurred by failing to account for small head motions in the *B* matrix, which 24 finds sufficient to disrupt tractography.

Although differences in DTI maps certainly arise between PGSE and STEAM acquisitions, we expect the similarity in principal directions to be similar enough to highlight gross errors introduced by incorrect modelling or lack of compensation.

The butterfly gradients affect the nominal *b* =0 images, as well as the diffusion-weighted images. In the absence of a strong diffusion gradient, the compensation counteracts the effect of crusher gradients, allowing additional echoes to affect the signal and leading to severe image artefacts. Thus the nominal *b* =0 images remain uncompensated with **G**_d_ = 0, but this is solved with a two-point fit to the non-normalized data. The *b* value in the nominal *b* =0 images defines the minimal diffusion weighting possible with sufficient crusher effect for the given image resolution. For example, in our *b* = 3425 s mm^− 2^ shell that value is 1316 s mm^− 2^. In the diffusion-weighted images, **G**_d_ works in principle as a crusher itself. In the absence of **G**_c_, the compensation must only correct for the contribution from **G**_s_.

## CONCLUSIONS

We demonstrate here that imaging gradients in the STEAM sequence can severely disrupt HARDI experiment design in a way not seen in PGSE and cause bias in parameter estimates. We introduce a compensation in the acquisition by simply subtracting the diffusion weighting of imaging gradients from the diffusion encoding gradients. This is simply implemented by updating the gradient scheme loaded on the scanner. Furthermore, models are presented to handle these problems retrospectively. These methods allow future work on and exploitation of the potential benefits of STEAM, especially for diffusion MRI on high-field scanners, where the lower effective *T*_2_ prevents long diffusion times in PGSE.

In particular, they enable us to evaluate STEAM ActiveAx for better sensitivity to large axons, which is the focus of our current work.

## Abbreviations

DTI: diffusion tensor imaging
DWI: diffusion-weighted imaging
DWS: diffusion-weighted spectroscopy
FA: fractional anisotropy
HARDI: high angular resolution diffusion imaging
PGSE: pulsed-gradient spin-echo
STEAM: stimulated echo acquisition mode
STEAMCOMP: compensated STEAM acquisition.
